# Supplementary material for: Binding of Amyloid β(1–42)-Calmodulin Complexes to Plasma Membrane Lipid Rafts in Cerebellar Granule Neurons Alters Resting Cytosolic Calcium Homeostasis
Source: Int J Mol Sci. 2021 Feb 17;22(4):1984. doi: 10.3390/ijms22041984 (PMC7923178; doi:10.3390/ijms22041984)
Supplement: Supplementary file 1 [file ijms-22-01984-s001.pdf]

Supplementary figures

IJMS_J.Poejo *et al*., 2021

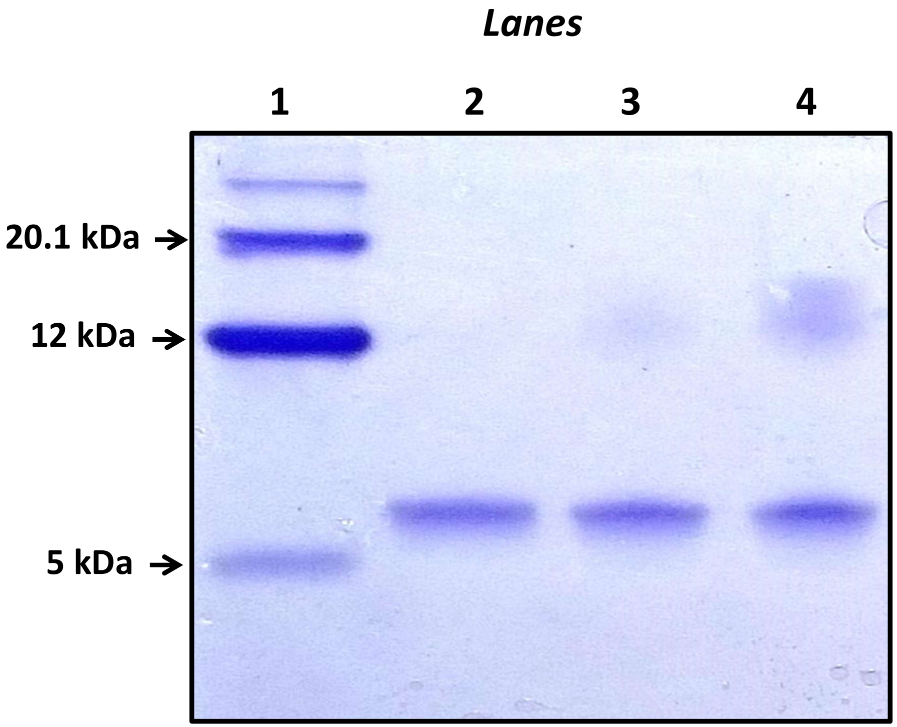

Supplementary Figure S1. Tricine-sodium dodecyl sulfate polyacrylamide electrophoresis of Aβ(1-42) solutions used in this work. Lanes: (1) Low molecular weight markers; (2) 177 µM Aβ(1-42) in phosphate-buffered saline (PBS) supplemented with 60 mM NaOH after preincubation during 2 hours at 37ºC; (3) 177 µM Aβ(1-42) in PBS after preincubation during 2 hours at 37ºC; (4) 177 µM Aβ(1-42) in PBS without preincubation at 37ºC. Molecular weight of the Aβ(1-42) monomer = 4.514 kDa.

Supplementary Figure S1 –J. Poejo *et al*_IJMS2021

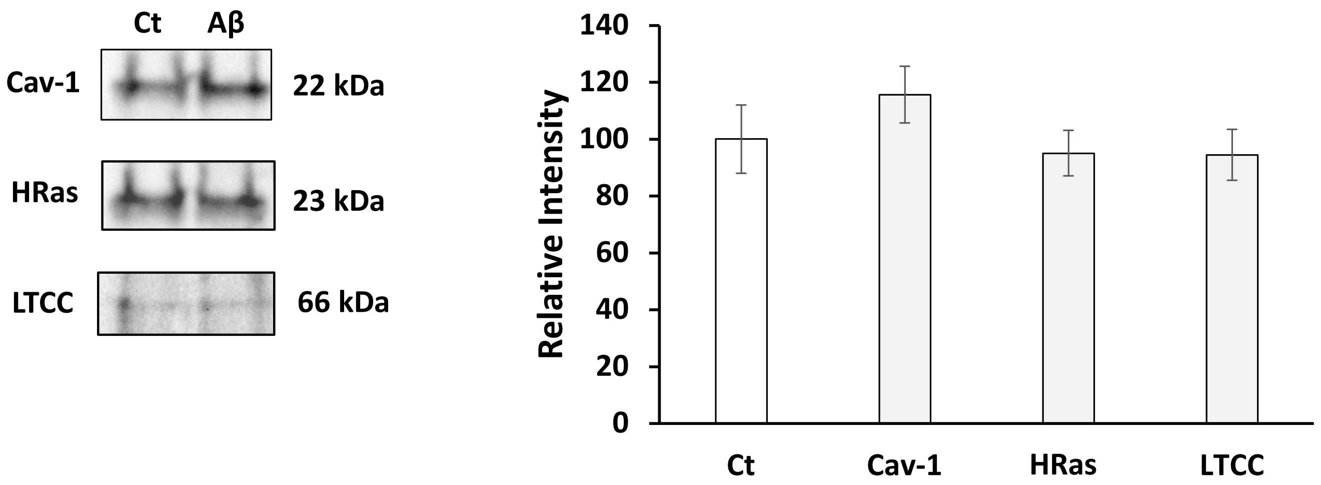

Supplementary Figure S2. **Western blotting of caveolin-1 (anti-Cav-1, sc 894), HRas (anti-HRas, sc 32026) and LTCC (anti-LTCC β subunit, sc 25689) after co-immunoprecipitation assay with mouse anti-β-amyloid antibody (Sigma, A8354) as described in the Materials and Methods**. The results shows that there is not a significant co-immunoprecipitation of these proteins by the anti-β-amyloid antibody.

Supplementary Figure S2 –J. Poejo *et al*_IJMS2021

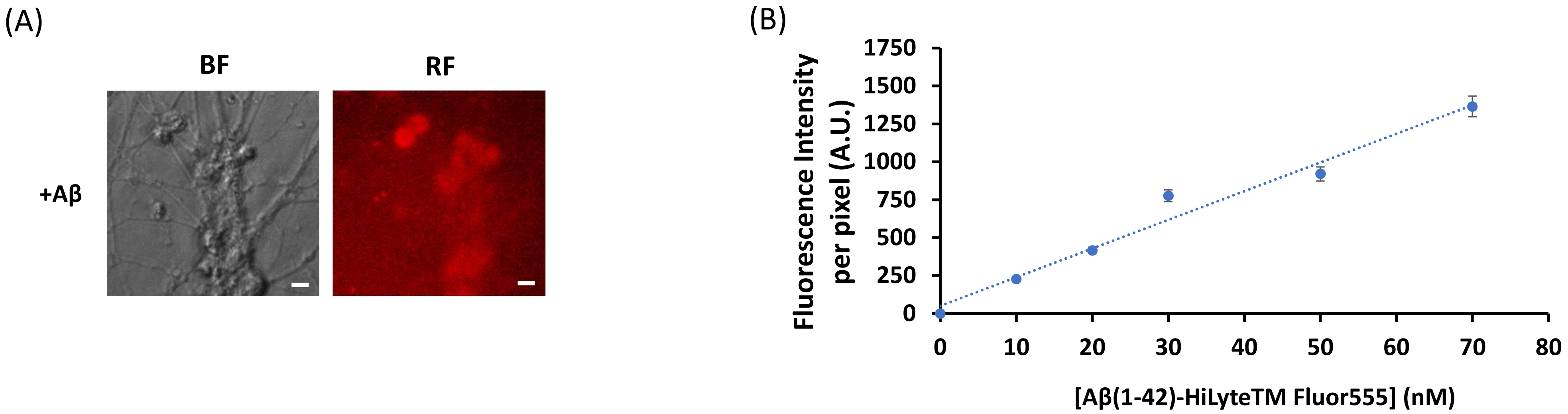

Supplementary Figure S3. **Internalization of Aβ(1-42)-HiLyte^{TM}-Fluor555 in mature CGN**. (**A**) Representative bright field (BF) and red fluorescence (RF) images of mature CGN after 2 hours incubation with 2 µM Aβ(1-42)-HiLyte^{TM}-Fluor555 (+Aβ) in Dulbecco's Modified Eagle Medium (DMEM) with 25 mM KCl acquired with an excitation filter of 556 nm and a dichroic mirror of 580 nm with emisión filter of 590 nm, and 0.03 s exposure time. No significant autofluorescence of CGN in the absence of Aβ(1-42)-HiLyte^{TM}-Fluor555 was detected over background with these experimental conditions and this short exposition time. After the 2 hours incubation of CGN with Aβ(1-42)-HiLyte^{TM}-Fluor555 the medium was replaced by MLocke's K25 and the Petri plate was placed in the holder of the fluorescence microscope thermostated at 37°C for images acquisition. Scale bar inserted in fluorescence microscopy images = 10 µm. (**B**) Calibration of the average intensity per pixel obtained with increasing concentrations of Aβ(1-42)-HiLyte^{TM}-Fluor555 in MLocke's K25 in the Petri plate. A.U. means arbitrary fluorescence units. Dotted line is the result of linear regression, R^2 = 0.9729. The average intensity readings per pixel in CGN neuronal somas were taken from several fields for a total number of 102 cells and after subtraction of CGN autofluorescence yielded an internalized concentration of 19.3 ±2.1 nM of Aβ(1-42)-HiLyte^{TM}-Fluor555 monomers by interpolation in the calibration line shown in the panel (**B**).

Supplementary Figure S3 –J.Poejo *et al*_IJMS2021

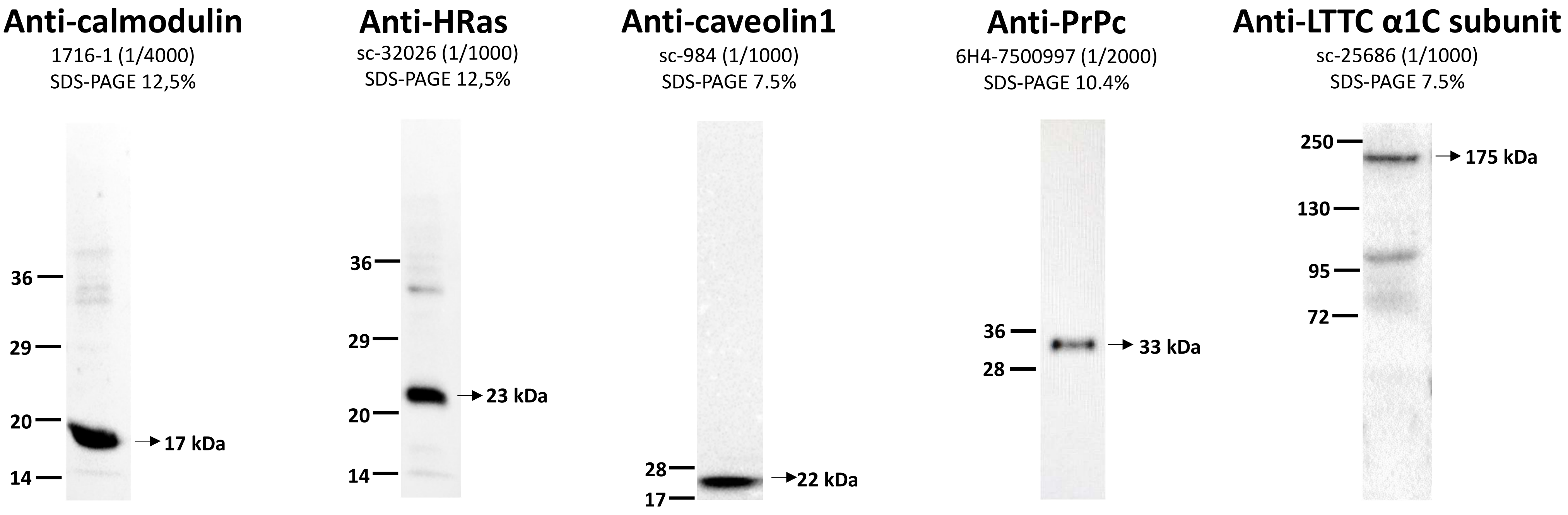

Supplementary Figure S4. Western blotting of CGN lysates with the primary antibodies used in fluorescence microscopy images. The reference number is included for each primary antibody, as well as the dilution used for the Western blotting and the percentage of acrylamide used to run the sodium dodecyl sulfate polyacrylamide electrophoresis (SDS-PAGE) in each case.

Supplementary Figure S4 –J. Poejo *et al*_IJMS2021
